# Experimental and Theoretical Studies on the Adsorption and Desorption Mechanisms of Chromate Ions on Cross-Linked Chitosan

**DOI:** 10.3390/jfb8040051

**Published:** 2017-12-14

**Authors:** Kenji Mishima, Xiaoyu Du, Shunsuke Sekiguchi, Naoki Kano

**Affiliations:** 1 Department of Chemical System Engineering, Graduate School of Engineering, The University of Tokyo, Tokyo 113-8656, Japan; 2 Graduate School of Science and Technology, Niigata University, 8050 Ikarashi 2-Nocho, Nishi-ku, Niigata 950-2181, Japan; F17K502A@mail.cc.niigata-u.ac.jp (X.D.); in.a.dream.over.282@gmail.com (S.S.); 3 Department of Chemistry and Chemical Engineering, Faculty of Engineering, Niigata University, 8050 Ikarashi 2-Nocho, Nishi-ku, Niigata 950-2181, Japan; kano@eng.niigata-u.ac.jp

**Keywords:** cross-linked chitosan, chromate ions, quantum chemistry calculation, pH dependence, adsorption, molecular geometries

## Abstract

In this work, chitosan bead materials were modified by cross-linking with epichlorohydrin (EP) and glutaraldehyde (GA) for the removal of heavy metals in wastewater. Using these cross-linked chitosan materials, the dependence of adsorption of chromate anions on pH was investigated experimentally and theoretically. The experimental results show that the adsorption process of the chromate (Cr) ions greatly depends on the pH of the solution, with the chitosan modified by cross-linking being an efficient adsorbent for chromate. On the other hand, quantum chemistry calculations were conducted to find out the factor determining the pH dependence of the adsorption efficiency of chromate ions on the dimer chitosan molecule, and show results similar to those found in the experiment. Both the experimental and numerical results show that the total charge numbers of the adsorbent and the adsorbate species and their relative molecular geometries are crucial in determining the adsorption efficiency.

## 1. Introduction

It is well known that investigations examining the abundant levels of toxic heavy metal ions discharged to the environment have now received considerable attention. One of the compelling reasons for this is the fact that these ions pollute air, soil, and water, thus having a great effect on human health [1]. It has now become an urgent agenda for humankind to protect the global environment and prevent hazardous effects on human bodies. Therefore, the development of effective methods to remove toxic metals from aqueous solutions has become necessary.

Cr (chromium) mainly exists in two oxidation states, Cr(III) and Cr(VI), in the natural aqueous environment. Cr(VI) has high toxicity and is a carcinogen, whose environmental standard is less than 0.05 mg/L and drainage standard is less than 0.5 mg/L. In the natural aqueous environment, it may be present in the form of CrO_4_^2−^ or HCrO_4_^−^. On the other hand, Cr(III) has low toxicity and is an essential material for living organisms, whose environmental standard is less than 2 mg/L. In the natural aqueous environment, it tends to form Cr(H_2_O)_n_(OH)_m_^(3−m)+^ and Cr(III)-organic complex. To remove hazardous heavy metals from polluted media, we can resort to several methods. These methods include evaporation, adsorption, chemical precipitation, ion exchange, and membrane separation. In particular, to remove heavy metals from aqueous solutions, the adsorption process has proven to be advantageous in view of technical and economical convenience [2,3]. It has been demonstrated that chitosan materials are some of the most promising adsorbents for the removal of chromium (Cr) species [4,5,6].

Furthermore, cross-linked chitosan bead materials are fabricated by GA (glutaraldehyde), which involves the reaction of the Schiff base between the aldehyde group of GA and the amines of chitosan. In this case, the amine groups and hydroxyl groups on the chitosan chain act as chelation sites for Cr. Cross-linked chitosan bead materials with EP (epichlorohydrin) are mainly associated with hydroxyl groups due to EP. Thereby, the original amino groups of chitosan are not affected or modified by cross-linking. These cross-linked chitosan materials are used to improve the adsorption behavior and to enhance the adsorption ability [7,8].

It has been verified that the adsorption process is most efficient at a pH of 4 and that all the adsorbents work more effectively in acidic regions compared to neutral and alkaline regions. In a low pH region, Cr(VI) exists in the form of negatively charged HCrO_4_^−^ in solution. As a result, electrostatic attraction between the negatively charged chromate ions and the positively charged groups of chitosan leads to an enhanced absorption capacity. EP contains amino (NH_2_) and hydroxyl (OH) groups [7,9], whereas GA contains hydroxyl (OH) groups. The latter can be protonated to yield ammonium ion (NH^3+^) in acidic regions [3], which helps to adsorb HCrO_4_^−^ by means of electrostatic attraction. However, the factor controlling the absorption capacity has not yet been identified experimentally.

Furthermore, quantum chemistry calculation is one of the most useful methods for investigating the microscopic behavior of adsorption mechanism among molecular species. In fact, some papers have applied quantum chemistry calculations to investigations examining the chemical properties of the interactions between metal ions and chitosan structures.

For example, DFT (density functional theory) calculations were conducted to study the interactions between the copper II ions and chitosan structures [3]. The geometries and the interaction energies of all the complexes were computed and the minimal energy conformations were retrieved. Zheng et al. performed quantum chemistry calculations to study the interactions between hexavalent chromium and *Sargassum* sp. during the biosorption [10]. They found that most of the absorbed Cr(VI) ions were reduced to Cr(III) after the biosorption, and they proposed a three-step removal mechanism of Cr(VI) by *Sargassum*.

Although some aspects of the adsorption mechanisms of metal ions on chitosan molecules that had not been clear experimentally have been clarified by the abovementioned theoretical works, it is important to investigate further. In essence, we will perform quantum chemistry calculations to analyze our experimental results microscopically and aim to search for dominant factors contributing to the adsorption efficiency, which will be a useful piece of information for designing good adsorbates in the future.

Therefore, in the present work, the Cr adsorption isotherm and kinetics are measured and discussed experimentally. Particular emphasis is placed on elucidating the effect of the pH of solution on the removal of chromate anions from both experimental and theoretical viewpoints. For this purpose, quantum chemistry calculation based on DFT is used to specify the most important factor controlling the pH dependence of the adsorption efficiency of chromate ions on the dimer chitosan molecules. From the calculation results, we found that the adsorption ability increases with an increase in the electrostatic attraction between the adsorbent and the adsorbate species, which is consistent with the experimental results. Finally, we conclude that the most important factor dominating the adsorption capacity is the total charge numbers of the adsorbent and the adsorbate species as well as their relative molecular geometries.

## 2. Experimental and Theoretical Details

### 2.1. Materials

Chemical reagents, including chitosan, was purchased from Tokyo Chemical Industry Co., Inc., Tokyo, Japan. Acetic acid, NaOH, EP, and GA were purchased from Kanto Chemical Industry Co., Inc., Tokyo, Japan, with all reagents used being of analytical grade. The water employed throughout the work (>18.2 MΩ) was treated by an ultrapure water system (RFU 424TA, Advantech Aquarius). The CrO_4_^2−^ standard solutions used for the calibration curve were prepared by diluting the standard solution (Kanto Chemical Co., Tokyo, Japan Inc., 1000 mg/L K_2_CrO_7_ solution). Our experimental solution was prepared to have concentrations of 0.05–5.0 mg/L by serial dilution from the stock solution of 1000 mg/L.

### 2.2. Preparation of Cross-Linked Chitosan Beads

Firstly, chitosan was stirred with 200 mL (2.0%) of acetic acid solution and added dropwise to 100 mL of 0.5 M NaOH [11,12].

Chitosan was added to 1.0 wt % of EP, with the pH adjusted to 14. After maintaining the mixed solution at 60 °C for 6 h, we obtained cross-linking with EP (Figure S1).

On the other hand, chitosan was added to 1.0 wt % of GA, with the pH adjusted to 7. After maintaining the mixed solution at room temperature for 24 h, we obtained cross-linking with GA (Figure S1).

SEM images for chitosan, EP, and GA are shown in Figures S2–S4, respectively. It is observed that the surfaces of EP and GA are rougher than that of chitosan. This may be due to the fact that the surface structures changed owing to the cross-linking reaction. Therefore, it seems that it may be a particulate.

### 2.3. Adsorption Experiments of Cr(VI)

The batch adsorption experiments of Cr(VI) were conducted as follows: kinetics of adsorption at room temperature were studied by adding 10–50 mg of adsorbents to a series of 100 mL conical flasks containing 0.05–5.0 mg/dm^3^ Cr(VI) solution at pH levels of 1–7, which was adjusted by 0.1 mol·dm^−3^ NaOH or HNO_3_. These flasks were moved to a shaker (130 rpm) for different periods of time (0.5–24 h) at 288–318 K.

Following each adsorption experiment, the cross-linked chitosan beads and the above Cr(VI) solution were filtered to remove Cr(VI) that had been adsorbed into the cross-linked chitosan beads. The concentration of Cr(VI) in the filtrate was determined by ICP-MS (inductively coupled plasma mass spectrometry).

The adsorption capacities of Cr(VI) using modified chitosan with GA and EP at equilibrium (qe: mg·g^−1^) were calculated using the following equation:

(1) $$q_{e}=\frac{\left(C_{i}-C_{e}\right)}{m}V$$

where *C_i_* and *C_e_* are the initial and equilibrium concentrations of Cr(VI) in a batch system, respectively (mg·L^−1^); *V* is the volume of the solution (L); and *m* is the weight of the adsorbent (g).

### 2.4. Quantum Chemistry Calculations

All of the quantum chemistry calculations were performed using the GAUSSIAN09 suite of programs [13]. The reactants and the reaction intermediates were fully optimized with Becke’s three-parameter hybrid functional coupled with the Lee-Yang-Parr correlation functional (B3LYP) level of theory and with the 6-31 G(d) basis set. To take into account the effect of the water environment on our target systems, the polarizable continuum medium (PCM method) with an appropriate dielectric constant of the solvent was used [14].

In order to apply the quantum chemistry calculations to cross-linked chitosan, it is effectively impossible to take into account the almost infinite chain length of the cross-linked chitosan. One of the assumptions we have made in the present calculations is that it can be represented by the chitosan dimer.

Through these calculations, we aimed to find the stable geometries of chitosan-chromate composites and to compare their relative energies. For these purposes, the following calculations were performed.

First, the chromate species (CrO_4_H_2_ and CrO_4_H^−^) and chitosan dimers were fully optimized separately. The sum of energies of the chromate species and the chitosan dimer can be thought of as the dissociation limit.

Second, the systems composed of one chromate species and one chitosan dimer were partially optimized. In that case, the atoms found in the neighborhood of the sites of attachment of the chromate species and the chitosan dimer were optimized through geometry, while those found further away were frozen. The motivation for this partial optimization is to take into consideration the assumption that the cross-linked chitosan will retain the original molecular structure when the adsorption or desorption process is taking place. Otherwise, the floppy structure of the chitosan dimer eases the dramatic structural modification of the chitosan dimer caused by the attachment of the chromate species, which is unrealistic in the case of the cross-linked chitosan.

## 3. Results and Discussion

**Figure 1:**
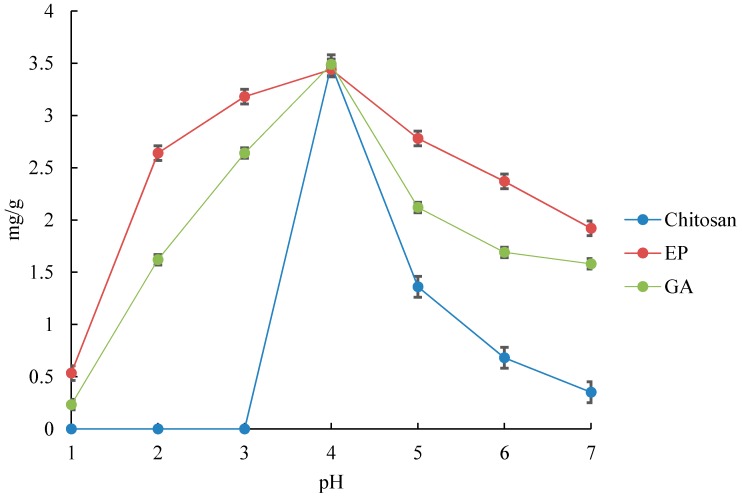
Effect of pH on the uptake of Cr(VI) by chitosan, EP, and GA. EP means “modified chitosan cross-linked with epichlorohydrin (EP)” and GA stands for “modified chitosan cross-linked with glutaraldehyde (GA)”. Error bars represent ±SD.

The experimental result of the effect of initial pH on modified chitosan is shown in Figure 1. During the experiment, the pH range was kept below 7.0 in order to avoid any bulk precipitation of Cr hydroxides.

It is well known that the chitosan molecules are protonated at the amino groups and are dissolved in the acidic region. This makes it impossible to perform adsorption experiments at pH levels of 1–3. However, EP and GA show large adsorption efficiency even in the acidic region. From these facts, it can be presumed that the chitosan monomers are cross-linked even in the acidic region, with the molecules adsorbed without any significant deformation of their molecular geometries. This is clearly demonstrated in Figure S5.

Figure 1 shows that GA has an adsorption efficiency larger than EP in the acidic region. This is due to the EP being cross-linked by hydroxyl groups, whereas GA is cross-linked by amino groups. Therefore, GA has fewer amino groups compared to chitosan and EP. From these results, it can be deduced that EP leads to the protonation of amino groups in the acidic region, whereas GA is not affected by protons because the adsorption by hydroxyl groups is dominant. Therefore, GA adsorbs more than EP, as shown in Figure 1.

In addition, Figure 1 shows that the adsorption efficiencies of all the chitosan molecules, GA, and EP deteriorate as the pH increases. As the chromates are negatively charged in any regions at all pH ranges, they ionically bond with the amino and hydroxyl groups of the chitosan molecules, GA, and EP, which are protonated. Therefore, it is presumed that in neutral and basic regions, the functional groups of the chitosan molecules, GA, and EP are not protonated due to of the decrease in the quantity of protons in solution, which leads to a decrease in the adsorption efficiency.

Furthermore, Cr(VI) exists in the form of CrO_4_^−^ and CrO_4_^2−^ at the pH range of 2–4, where CrO_4_^−^ is dominant. On the other hand, CrO_4_^2−^ becomes increasingly dominant as the solution becomes more basic, and the form of CrO_4_^2−^ becomes stable above the pH of 7. The amino groups of the adsorbent become deprotonated as the pH increases. From these facts, the functional groups that combine with chromate ions decrease because their ion valence increases. Therefore, as shown in Figure 1, the adsorption efficiencies of all the chitosan molecules, GA, and EP deteriorate as the pH increases above 7.

From Figure 1, it can be seen that the uptake of Cr(VI) is effective even at the pH range of 1–3 by cross-linking, and that the adsorption capacity of Cr(VI) reaches its maximum at a pH of 4 for the adsorbents, which is due to the changes in the surface charge of the adsorbent. At a lower pH, the surface of the adsorbent may become protonated and more positively charged, which would attract the chromate anions more. At a higher pH, the hydroxyl ions in the solution may combine with chromate ions to form precipitates. The pH of the aqueous solution can affect the surface charge of the adsorbent, the degree of ionization, speciation of metal ions, and surface metal binding sites.

**Figure 2:**
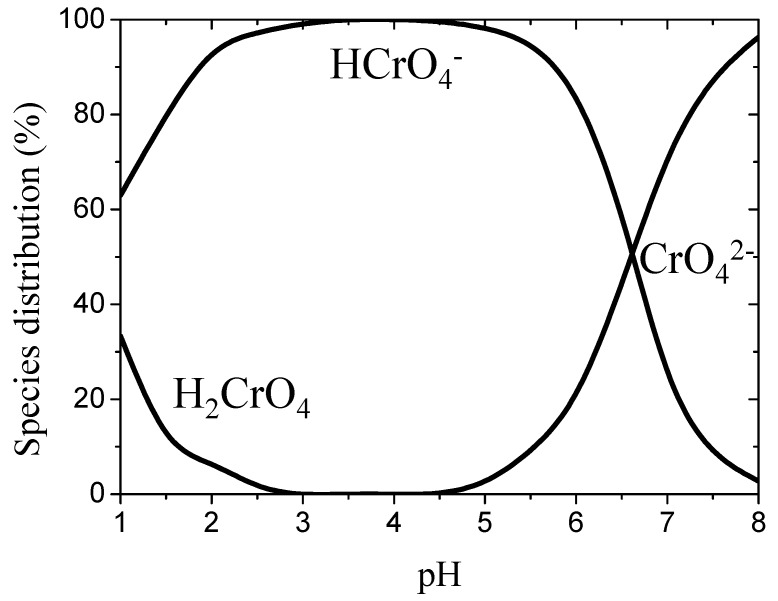
Species distribution of Cr(VI).

On the other hand, the dependence of hydrogen chromate species distribution on pH is shown in Figure 2. It can be seen that Cr(VI) exists as a neutral chromic acid (H_2_CrO_4_) in the pH range of 1–2; as hydrogen chromate anions (HCrO_4_^−^) in the pH range of 2–6.5; and as chromate anions (CrO_4_^2−^) above a pH of 6.5. It was shown that the species of Cr(VI) depends on the pH of solution and total chromate concentration [15]. It was observed that the uptake of Cr(VI) decreases with an increase in pH. This may be attributable to the larger amount of OH^−^ ions present in the mixture, which competes with Cr(VI) species [16]. On the other hand, NH_2_ groups are deprotonated and form negatively charged sites as pH increases. As a result, the electrostatic repulsion between negatively charged sites and negatively charged hexavalent chromium ions makes it difficult for Cr(VI) to adsorb [17]. Therefore, it may be reasonable to formulate the adsorption reactions by the following equations:

HCrO_4_^−^↔CrO_4_^2−^ + H^+^  pKa = 5 (2)

H_2_CrO_4_↔HCrO_4_^−^ + H^+^  pKa = 4.1 (3)

Cr_2_O_7_^2−^ + H_2_O↔2 HCrO_4_^−^  pKa = 2.2 (4)

Therefore, we have verified that the adsorption process of the chromate ions greatly depends on the pH of the solution, as shown in Figure 2, which is consistent with the results of Laus et al. [1]. To clarify the origin of this phenomenon, we performed quantum chemistry calculations as shown below. We assumed that the chromate species was CrO_4_H_2_ and CrO_4_H^−^ in the low (1–3) and the middle (3–7) pH regions, respectively, with the chitosan dimers being singly or doubly protonated on the amino groups.

**Figure 3:**
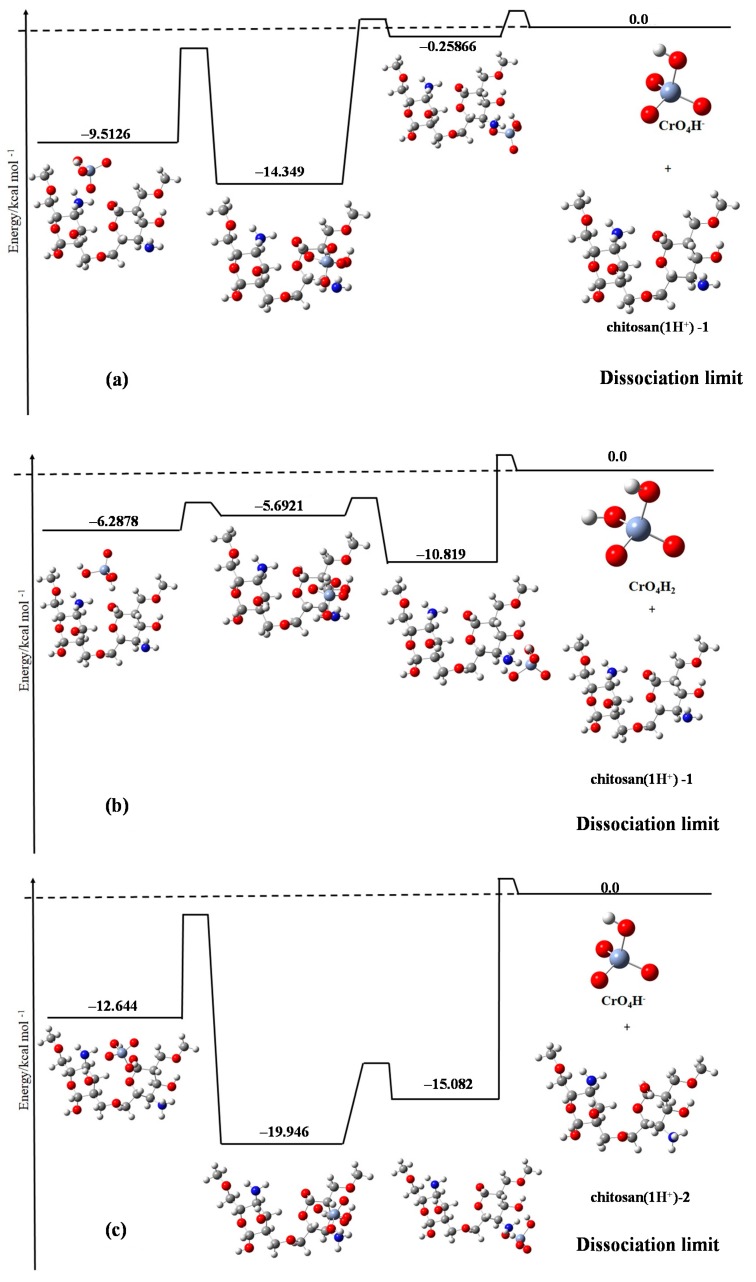

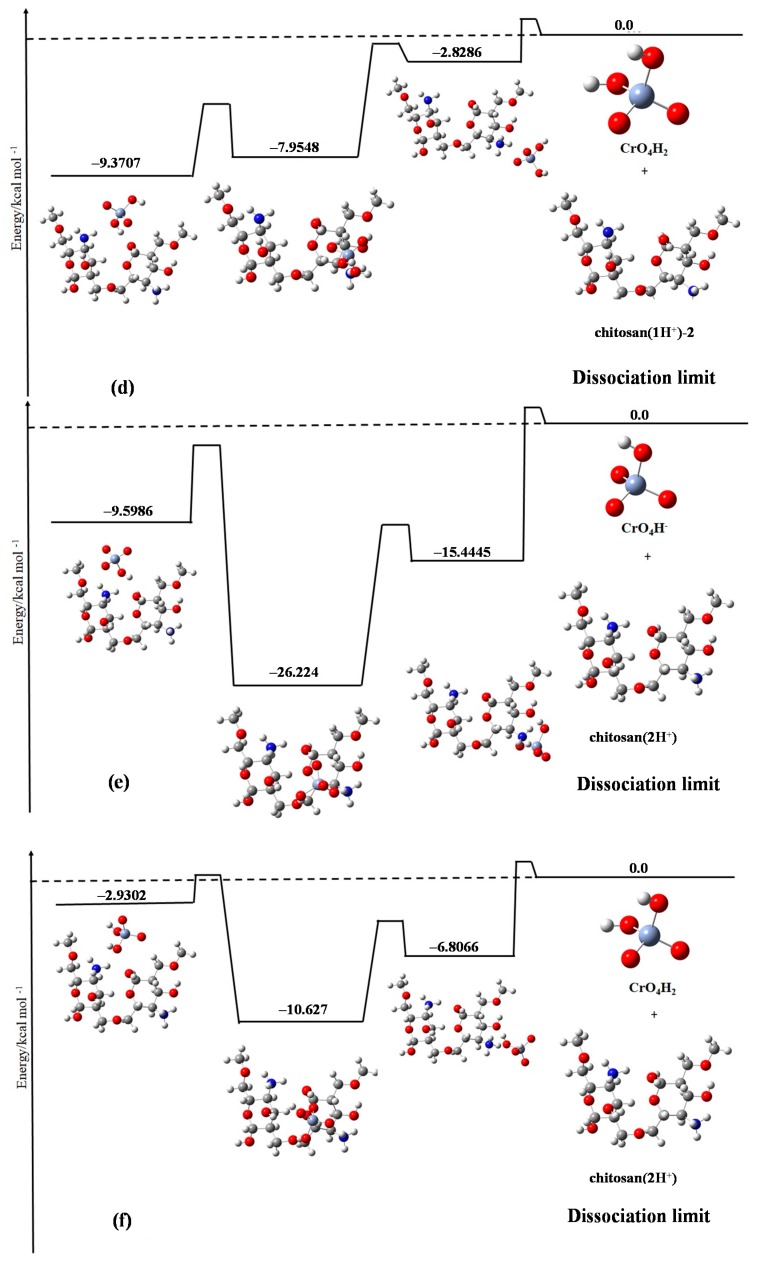
(**a**) CrO_4_H^−^ and chitosan (1 H^+^)-1; (**b**) CrO_4_H_2_ and chitosan (1 H^+^)-1; (**c**) CrO_4_H^−^ and chitosan (1 H^+^)-2; (**d**) CrO_4_H_2_ and chitosan (1 H^+^)-2; (**e**) CrO_4_H^−^ and chitosan (2 H^+^); and (**f**) CrO_4_H_2_ and chitosan (2 H^+^). The symbol chitosan (1 H^+^)-1 means that one proton is attached to one of the nitrogen atoms of chitosan dimer, while chitosan (1 H^+^)-2 means that one proton is attached to the other nitrogen atom of the chitosan dimer. The symbol chitosan (2 H^+^) implies that two protons are attached to both the nitrogen atoms of the chitosan dimer. In the extremely low pH region, (**f**) is the most probable, while in the middle pH region, (**a**,**c**) are the most probable. The other cases may depend on the experimental setup. The white, grey, blue, red, and bluish grey circles represent H, C, N, O, and Cr atoms, respectively.

Figure 3 shows the six cases considered in our calculations, with the calculation details shown in the caption.

The most impressive conclusion drawn from these figures is that the complex of chromate species and chitosan dimer with chromate ions being CrO_4_H^−^ is more stable than the case with chromate being the neutral CrO_4_H_2_. This is due to the fact that the electrostatic interaction between the molecules have a greater opposite charge (Figure 3a,c), which is stronger than that between the molecules with a smaller opposite charge (Figure 3f). In particular, Figure 3e shows the largest stabilization energy compared to any other panels. In this case, the chromate ion is CrO_4_H^−^ with two protons attached to the chitosan dimer, which possibly could have the strongest electrostatic attraction between the adsorbate and the adsorbent. This is consistent with the experimental observation that in the extremely low pH region (1–3), the adsorption efficiency deteriorates.

The second finding is that in almost all of the cases, the complex composed of the adsorbent and the adsorbate has the largest stabilization energy when the chromate species is attached face-on on the chitosan dimer compared to any other molecular configurations. This may be due to the more efficient orbital interaction (or orbital hybridization) in the former case. This is one of the mechanisms of stabilizing the adsorbent-adsorbate composites that was not found through the experimental results.

By combining the experimental and theoretical results, it was found that the most important factor controlling the pH dependence of the adsorption efficiency of chromate species on the dimer chitosan molecules is the total charge numbers of the adsorbent and the adsorbate species as well as their relative molecular geometries.

Finally, because the room temperature (~300 K) corresponds to 0.60 kcal/mol, it is difficult for all of the cases shown in Figure 3 to overcome the potential barrier to return to the dissociation limit. This means that the adsorption efficiency of the cross-linked chitosan is relatively good for the chromate species in the low pH region. This is also consistent with the experimental evidence.

## 4. Conclusions

In conclusion, our experimental results show that the adsorption of Cr(VI) onto cross-linked chitosan beads is highly dependent on pH and that the uptake of Cr(VI) is effective even at the pH range of 1–3 by cross-linking. The adsorption capacity of Cr(VI) reaches its maximum at a pH of 4 for the adsorbents. However, the amount of Cr(VI) adsorption onto EP is higher than that of chitosan and GA at the same pH. All of the above results demonstrate that chitosan modified by cross-linking can be an efficient adsorbent for Cr.

The quantum chemistry calculations revealed that the total charge numbers of the adsorbent and the adsorbate and their relative molecular geometries are crucial in determining the adsorption efficiency.

Combining the experimental and theoretical results, we propose that one of the strategies to develop a new highly efficient adsorbent-adsorbate combination is to maximize the difference between the total charge of the adsorbent and that of the adsorbate.

## Supplementary Materials

The following are available online at www.mdpi.com/2079-4983/8/4/51/s1, Figure S1; Synthesis of EP and GA, Figure S2; Secondary electron image by SEM of chitosan surface structure before adsorption, Figure S3; Secondary electron image by SEM of EP surface structure before adsorption, Figure S4; Secondary electron image by SEM of GA surface structure before adsorption, Figure S5; Molecular structures of chitosan, EP, and GA.

## References

[1] Laus R., Costa T.G., Szpoganicz B., Fávere V.T. (2010). Adsorption and Desorption of Cu(II), Cd(II) and Pb(II) Ions Using Chitosan Crosslinked with Epichlorohydrin-triphosphate as the Adsorbent. J. Hazard. Mater..

[2] Huang G., Zhang H., Shi J.X., Langrish T.A.G. (2009). Adsorption of Chromium(VI) from Aqueous Solutions Using Cross-Linked Magnetic Chitosan Beads. J. Ind. Eng. Chem. Res..

[3] Wan Ngah W.S., Ghani S.A., Kamari A. (2005). Adsorption Behaviour of Fe(II) and Fe(III) Ions in Aqueous Solution on Chitosan and Cross-linked Chitosan Beads. Bioresour. Technol..

[4] Modrzejewska Z., Sujka W., Dorabialska M., Zarzycki R. (2006). Adsorption of Cr(VI) on Cross-linked Chitosan Beads. Sep. Sci. Technol..

[5] Jaros K., Kaminski W., Albinska J., Nowak U. (2005). Removal of Heavy Metal Ions: Copper, Zinc and Chromium from Water on Chitosan Beads. Environ. Prot. Eng..

[6] Zarzycki R., Sujka W., Dorabialska M., Modrzejewska Z. (2002). Adsorption of Cr(VI) on Chitosan Beads. Chem. Inz. Ekol..

[7] Ngah W.S., Endud C.S., Mayanar R. (2002). Removal of Copper(II) Ions from Aqueous Solution onto Chitosan and Cross-linked Chitosan Beads. React. Funct. Polym..

[8] Cestari A.R., Vieira E.F.S., Oliveira I.A., Bruns R.E. (2007). The Removal of Cu(II) and Co(II) from Aqueous Solutions using Cross-linked Chitosan-evaluation by the Factorial Design Methodology. J. Hazard. Mater..

[9] Chiou M.S., Li H.Y. (2003). Adsorption Behavior of Reactive Dye in Aqueous Solution on Chemical Cross-linked Chitosan Beads. Chemosphere.

[10] Zheng Y.-M., Liu T., Jiang J., Yang L., Fan Y., Wee A.T.S. (2011). Characterization of Hexavalent Chromium Interaction with Sargassum by X-ray Absorption Fine Structure Spectroscopy, X-ray Photoelectron Spectroscopy, and Quantum Chemistry Calculation. J. Colloid Interface Sci..

[11] Ngah W.S.W., Teong L.C., Hanafiah M.A.K.M. (2011). Adsorption of Dyes and Heavy Metal Ions by Chitosan Composites: A Review. Carbohydr. Polym..

[12] Sushanta D., Arjun M., Kriveshini P. (2014). Magnetic Chitosan–GO Nanocomposite: Synthesis, Characterization and Batch Adsorbed Design for Cr(VI) Removal. J. Environ. Chem. Eng..

[13] Frisch M.J., Trucks G.W., Schlegel H.B., Scuseria G.E., Robb M.A., Cheeseman J.R., Scalmani G., Barone V., Petersson G.A., Nakatsuji H. (2009). GAUSSIAN09.

[14] Tomasi J., Mennucci B., Camm R. (2005). Quantum Mechanical Continuum Solvation Models. Chem. Rev..

[15] Wu Y., Luo H.J., Wang H., Wang C., Zhang J., Zhang Z.L. (2013). Adsorption of Hexavalent Chromium from Aqueous Solutions by Graphene Modified with Cetyltrimethylammonium Bromide. J. Colloid Interface Sci..

[16] Heidari A., Younesi H., Mehraban Z. (2009). Removal of Ni(II) Cd(II), and Pb(II) from a ternary aqueous solution by amino functionalized mesoporous and nano mesoporous silica. Chem. Eng. J..

[17] Adamczuk A., Kołodyńska D. (2015). Equilibrium Thermodynamic and Kinetic Studies on Removal of Chromium, Copper, Zinc and Arsenic from Aqueous Solutions onto Fly Ash Coated by Chitosan. Chem. Eng. J..

