# Supplementary material for: Experimental and Theoretical Studies on the Adsorption and Desorption Mechanisms of Chromate Ions on Cross-Linked Chitosan
Source: J Funct Biomater. 2017 Dec 14;8(4):51. doi: 10.3390/jfb8040051 (PMC5748558; doi:10.3390/jfb8040051)
Supplement: Supplementary file 1 [file jfb-08-00051-s001.pdf]

Sopporting Information: Experimental and Theoretical Studies on the Adsorption and Desorption Mechanisms of Chromate Ions on Cross-Linked Chitosan

Kenji Mishima , Xiaoyu Du, Shunsuke Sekiguchi and Naoki Kano

1. Synthesis of EP and GA

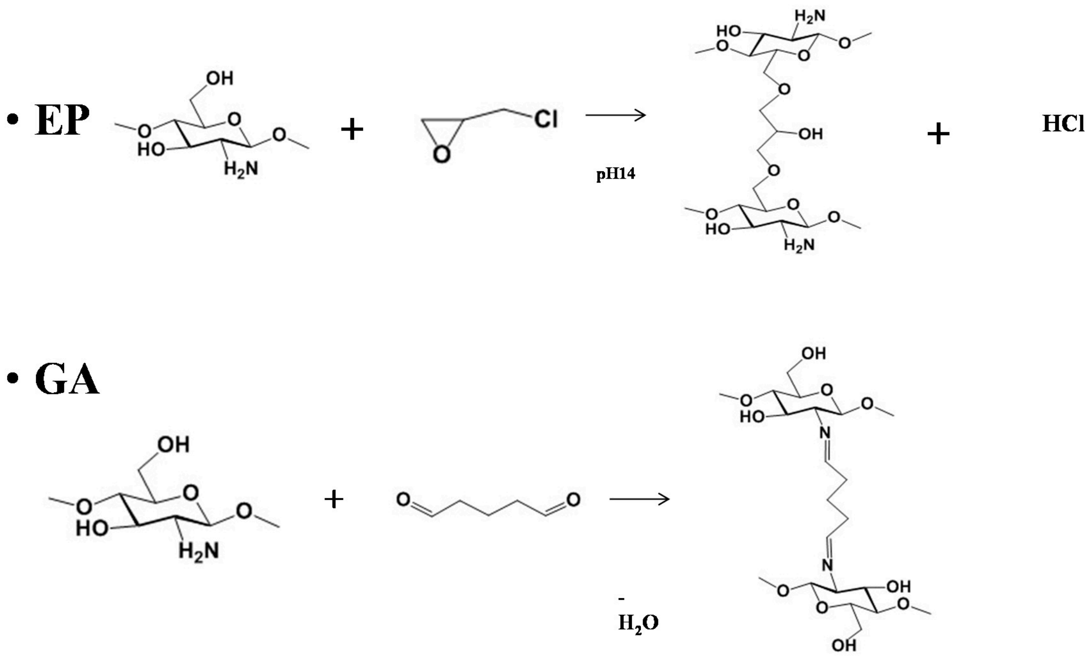

Fig.S1 Synthesis of EP and GA

2. Secondary electron images by SEM of chitosan, EP, and GA surface structures

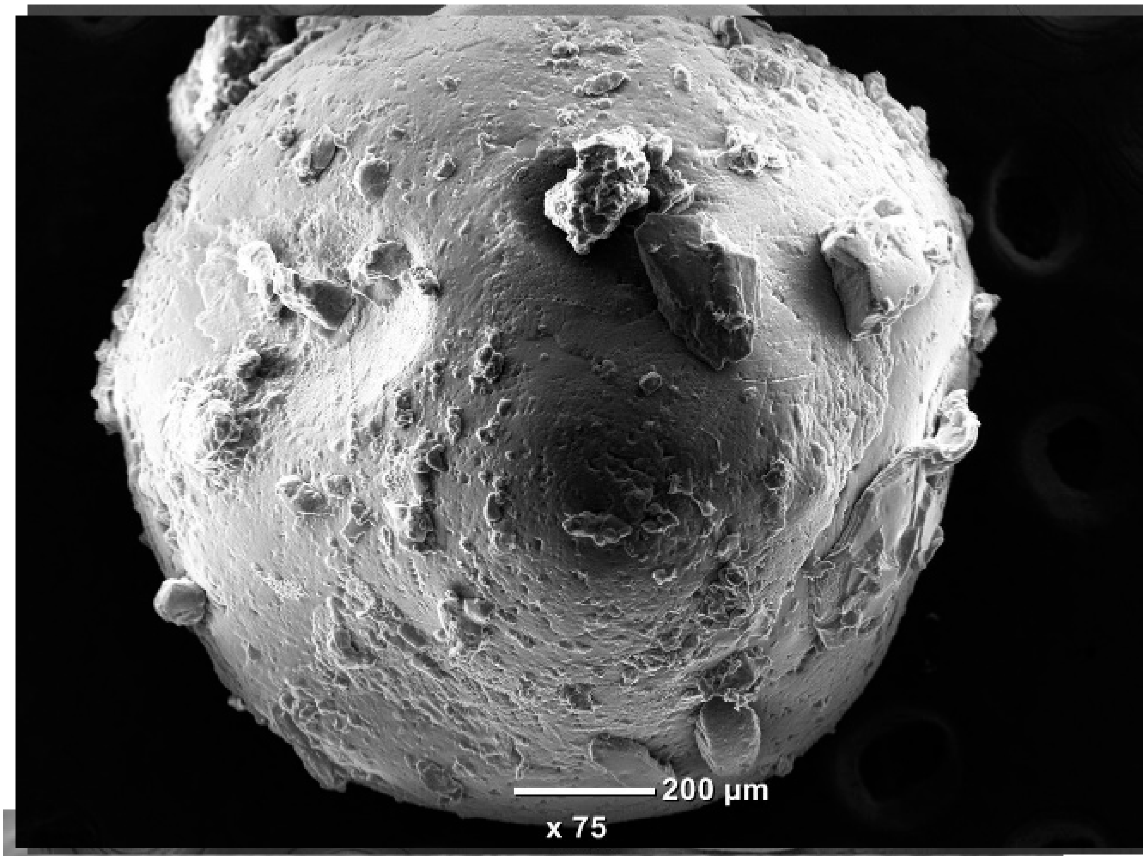

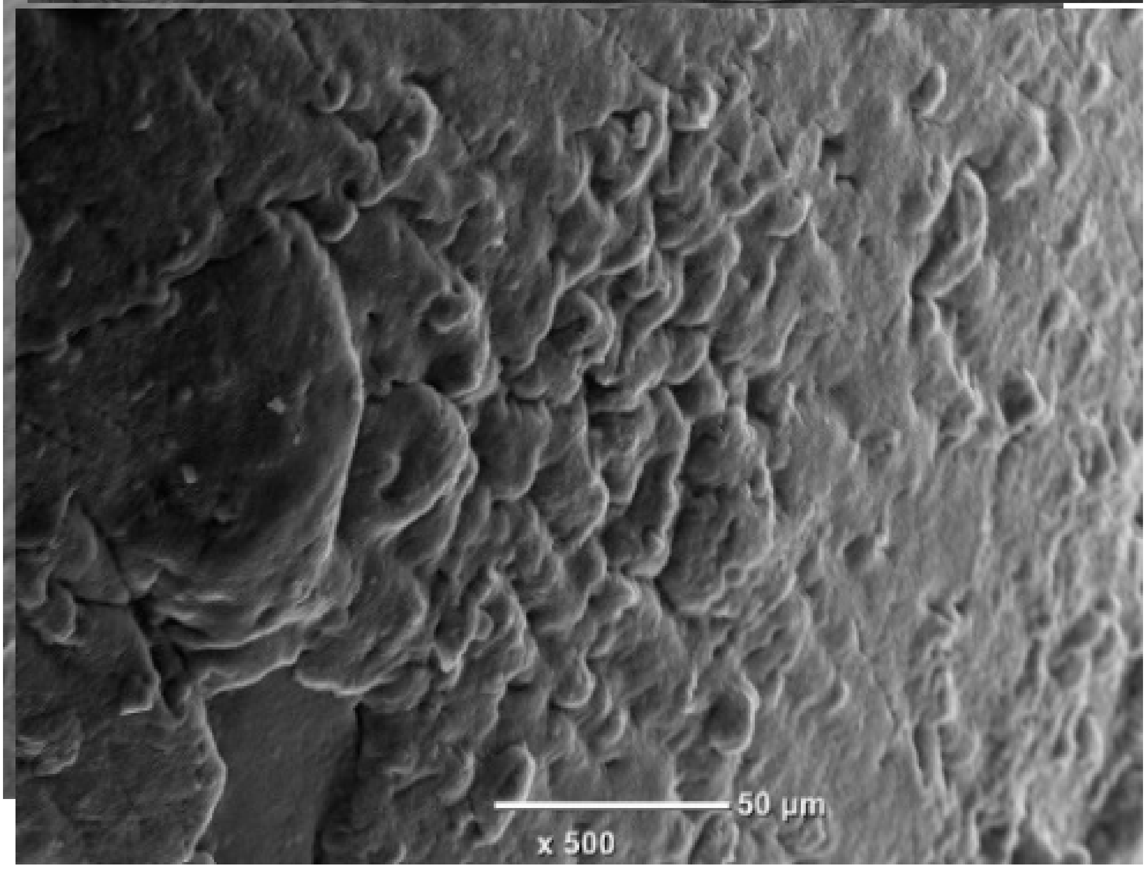

Fig.S2 Secondary electron image by SEM of Chitosan surface structure before adsorption.

Fig.S3 Secondary electron image by SEM of EP surface structure before adsorption.

Fig.S4 Secondary electron image by SEM of GA surface structure before adsorption.

3. molecular structures of chitosan, EP, and GA

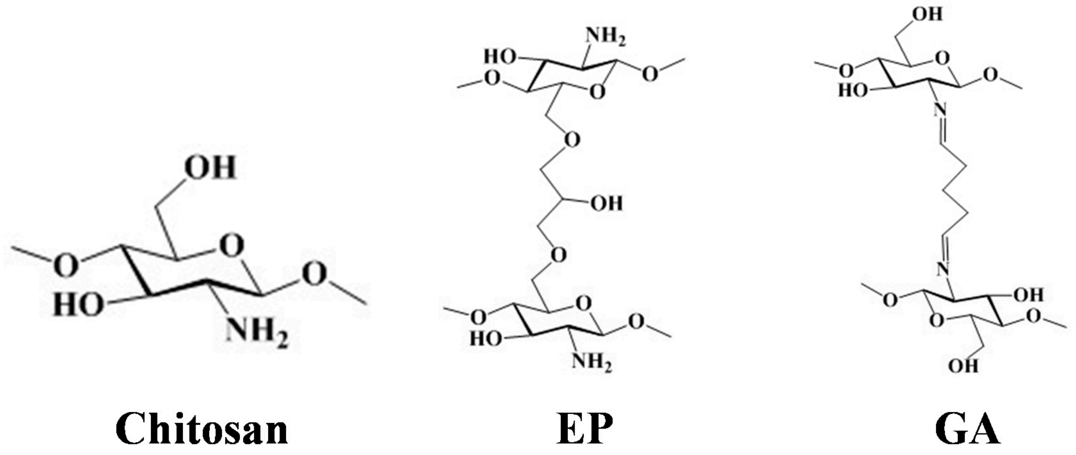

Fig.S5 Molecular structures of chitosan, EP, and GA
